# Knowledge of Blood Transfusion in Medical And Biology Students

**DOI:** 10.7759/cureus.6133

**Published:** 2019-11-12

**Authors:** Maria Moschidou, Irene P Tzanetakou, Demetris Lamnisos, Eftychia Kontekaki, Zacharias Fasoulakis, Emmanuel N Kontomanolis

**Affiliations:** 1 Miscellaneous, Democritus University of Thrace, Alexandroupolis, GRC; 2 Epidemiology, European University Cyprus, Nicosia, CYP; 3 Miscellaneous, Democritus University of Thrace, University Hospital of Alexandroupolis, Alexandroupolis, GRC; 4 Obstetrics and Gynecology, National and Kapodistrian University of Athens, Athens, GRC; 5 Obstetrics and Gynecology, Democritus University of Thrace, Alexandroupolis, GRC

**Keywords:** knowledge, blood transfusion, blood products, medical - biology students, infectious - contagious diseases

## Abstract

Introduction: Blood transfusion (hemotherapy) is a therapeutic intervention used in treatment strategies of multiple diseases, thus, proper education is of utmost importance. Since currently there are no specified educational programs, undergraduate students were evaluated for the knowledge gained during university courses.

Purpose: To evaluate and compare the level of knowledge of students of the faculty of Health Science, Department of Medicine (DM), and Department of Molecular Biology-Genetics (DMB&Gs) on issues related to the transfusion of blood products.

Methods: A cross-sectional observational study was carried out with 123 students from the aforementioned departments of the Democritus University of Thrace, from the third year to the last year of study. A questionnaire was used, weighted, and was based on the European Commission's Guide to the Preparation, Use and Quality Assurance for Blood Components. Statistical tests such as chi-square (χ2), t-test, analysis of variance (ANOVA), and linear regression were used to investigate the factors that affect the overall score.

Results: The mean score of the students was 42.55 while the standard deviation (SD) was 12.27. The difference in the scores between the students of the DM (M = 44.63, SD = 13.2) and those of the DMB&Gs (mean = 38.25, SD = 9.05) was statistically significant in the univariable analysis (t= 3.1, p = 0.0), but in the multivariable analysis, it was not statistically significant (β = -4.1, p = 0.1.). The results of the multiple regression model indicated that the year of study, the professional status of the father, and the grade in the hematology course were associated with the total score.

Conclusions: The level of knowledge regarding blood product transfusion among students of the faculty of Health Science is insufficient.

## Introduction

Blood transfusion (hemotherapy) is a therapeutic intervention and an important part of modern healthcare. According to the World Health Organization (WHO), transfusion is the process of transferring blood or blood products derived from a donor into the vasculature and subsequently the circulating blood of the recipient, carried out by inserting an intravenous needle or catheter in the patient and followed by administration of blood or blood products [1]. Blood transfusion is considered to be the first successful organ transplant [2-3]. Blood is a "fluid tissue" consisting of plasma and cellular components (red blood cells, white blood cells, platelets) that circulates through the vascular system throughout the body [2]. Hemotherapy was ranked the first among medical treatment interventions in the US in 2011, showing an increase of 134%, from 1997 to 2011 [4]. In 2012, the American Medical Association (AMA) and the Joint Commission ranked blood transfusion as the fifth most common treatment worldwide, associated with an increased risk of serious and undesirable complications [5]. The choice of the appropriate blood product, at the right time, and the appropriate indication determines its success [1,6]. For the rational use of blood, special knowledge is required [5].

Hemovigilance data worldwide report mistakes at all stages of transfusion which results in cases involving risks and serious complications for the patient [7]. The risks are transfusion-transmitted infections, sepsis, haemolytic reactions, acute pulmonary damage, bacterial infection, allergic reaction, circulatory overload, etc. [8-10]. Since the establishment of hemovigilance in the UK in 1996, analysis has been performed in more than 2,800 serious adverse reactions associated with incorrect blood transfusion, 27 deaths, and 596 cases of patients with significant morbidity [8].

In Greece, according to data from the National Center for Blood Donation, every year, 550,000-700,000 units of blood are transfused, in thalassemic, haematological, and oncology patients undergoing surgical procedures and in generalized haemorrhages; 120,000 of these units cover those suffering from thalassemia [5,11]. To ensure the optimal and correct use of blood transfusions, there must be thorough training of clinical doctors, nurses, and biologists that perform serological tests in medical laboratories such as hepatitis C virus (HCV), human immunodeficiency virus (HIV), hepatitis B virus (HBV), and rapid plasma reagin (RPR). Studies carried out in various countries have shown that medical students have insufficient knowledge about transfusions [12-14]. The conclusion was that their education should be enhanced [3,15-16]. The United Kingdom stressed that each member of the staff involved in the transfusion process should have access to training regarding their role [8,17]. Mark Friedman (2011) considered that the lack of knowledge of clinicians is an obstacle to good transfusion practices and he suggested that students be properly trained [18].

The purpose of this study was to examine, evaluate, and relate the knowledge of the students of the faculty of Health Sciences, Department of Medicine (DM), and Department of Molecular Biology-Genetics (DMB&Gs) regarding hemotherapy since their participation is of major importance for the transfusion process.

## Materials and methods

Study sample

The study involved 123 students from DM and DMB&Gs of the Democritus University of Thrace, who were in the third to the last year of study and attended the course of hematology-immunology. The data was collected from May to June, 2017; 82 were students of DM, 40 were students of DMB&Gs and one student who did not mention his department. Convenience sampling was used. For the qualitative and quantitative measurement of the variables, the questionnaire used was made up of two parts; it was developed for the purposes of the survey in accordance with the guidelines of preparation, use, and quality assurance of blood components of the European Commission. The first contains social-demographic characteristics questions of the sample and individual information related to the research topic. The second part (special questionnaire) contains multiple-choice questions and refers to the following: terms relating to blood products transfusion, their safe collection, maintenance and administration, and blood-borne infections. A pilot study with a small number of participants (n = 15) was used to weigh the questionnaire. More specifically, the second part includes questions about the transfusion process, blood components, and how each one is transfused, the ABO blood group system, and other specialized questions regarding the maintenance of blood components and potential side effects from the blood transfusion process. Responses were positive for its understanding, acceptance, and composition, while the average completion time was about 10 minutes (Table 1).

**Table 1:** Results of the multiple-choice questionnaire on blood products transfusion

Variable	Frequency (%) n=123
A. What is the maximum transfusion time of concentrated red blood cells?	
Between 2 - 3 hours	20 (17,09)
Between 1 - 5 hours	71 (60,68)
Until 4 hours	17 (14,53)
Until 5 hours	9 (7,69)
B. How long after departure from hematology department, should platelets be transfused?	
As soon as possible	82 (68,91)
Within an hour	24 (20,17)
Depending on the patient’s health status	13 (10,92)
C. During transfusion, the patient develops fever with or without quiver. What actions will you take?	
Immediate stop of blood transfusion	33 (26,83)
Venous line preservation	3 (2,44)
Vital Signs Control	2 (1,63)
Complete an “Event Report”	4 (3,24)
Blood Bank Update	2 (1,63)
All of the above	79 (64,23)

Statistical analysis

In order to describe the four factors (blood groups, blood products, blood transfusion, and infectious diseases) and the description of the quantitative variables, the numerical summaries of the mean and standard deviation (SD), the minimum and the maximum score observed were reported, while for the categorical variables the frequency and the relative frequency are presented. The independent samples t-test and analysis of variance (ANOVA) statistical tests were used to investigate the relationship between: (a) demographic data with total score (score 0-100); and (b) correlation of responses to blood transfusion issues with the overall score (score 0-100). The χ2 statistical test was used to examine whether the categorical variable with two categories (<50 or >50 scores) is related to demographic characteristics. The multiple regression model was used to examine the relationship between the statistically significant variables in the univariable analysis and the overall score (score 0-100). The statistical analysis was performed with Statistical Package for the Social Sciences (SPSS) for Windows, version 20, (SPSS Inc., Chicago, IL). and the statistical level of significance was set at p<0.05.

## Results

Of the 123 students participated in the study, 58 were male (47.15%) and 65 were female (52.85%). A total of 113 students were <25 years old, while four students were >25 years old. Regarding the educational level of the students' fathers, 50.82% are university graduates, 34.43% high school graduates, and 14.75% technological institutes graduates, while regarding their professional situation 36.67% are civil servants, 30% self-employed, 16.67% employees, 14.17% pensioners, and 2.5% unemployed. The results of the independent samples t-test indicated that students of DM had a statistically significant higher score (mean= 44.63, SD= 13.19) than the students of DMB&Gs (mean = 38.25, SD= 9.05). The results of ANOVA test indicated that the year of study was related with the overall score (p< 0.05). The highest score was observed in the fifth year of studies (mean= 50.92, SD= 13.53), followed by the sixth year (mean = 48.00, SD= 11.16), the fourth year (mean = 42.46, SD= 11.11) and finally the third year (mean = 35.79, SD= 8.49). The overall score was affected by the grade in the course of hematology-immunology (p <0.05). The highest score was observed at 8-8.9 (mean= 54.40, SD= 9.43), followed by a score of 9-10 (mean = 51, SD= 12.19), a score of 5-6.9 (mean = 38.06, SD = 11.63) and a score of 7-7.9 (mean = 37.89, SD = 8.15) (Table 2).

**Table 2:** Association of total grade/score (0-100) with independent variables (frequency, relative frequency, arithmetic mean, standard deviation, p-value) * t-test, + analysis of variance (ANOVA) test.

Variable	Frequency (%) n=123	Mean score (sd)	P - value
Sex			
Male	58 (47.15)	43.66(12.69)	*0.35
Female	65 (52.85)	41.77(11.90)	
Age			
<25 years	113 (96.58)	42.90(11.94)	*0.33
>25 years	4 (3.42)	52.00(15.68)	
Department/School			
Medicine	82 (67.21)	44.63(13.19)	*0.01
Biology	40 (32.79)	38.25(9.05)	
Graduate of another school			
Yes	3 (2.73)	51.00(22.52)	*0.59
No	107 (97.27)	42.64(12.28)	
Year of Study			
3th	34(31.78)	35.79(8.49)	+0.00
4^th^	26(24.30)	42.69(11.11)	
5^th^	25(23.36)	50.92(13.53)	
6^th^	22(20.56)	48.00(11.16)	
Father's education level			
Senior High school	42(34.43)	40.26(11.58)	+0.20
Technological Institute	18(14.75)	46.39(11.81)	
University Degree	62(50.82)	43.00(12.81)	
Mother's education level			
Senior High school	44(36.36)	40.80(10.77)	+0.48
Technological Institute	24(19.83)	42.83(12.77)	
University Degree	53(43.80)	43.85(13.45)	
Father's professional status			
Employee	20(16.67)	42.55(11.53)	+0.25
Civil Servant	44(36.67)	40.27(11.08)	
Self-employed	36(30.00)	42.97(14.50)	
Pensioner	17(14.17)	48.18(11.63)	
Unemployed	3(2.50)	38.00(9.00)	
Mother's professional status			
Employee	32(26.02)	40.38(12.78)	+0.10
Civil Servant	47(38.21)	41.96(11.33)	
Self-employed	16(13.01)	44.44(14.39)	
Pensioner	10(8.13)	52.10(10.57)	
Unemployed	18(14.63)	41.00(11.14)	
Grade in the hematology course			
5-6,9	32(34.04)	38.06(11.63)	+0.00
7-7,9	47(50.00)	37.89(8.15)	
8-8,9	10(10.64)	54.40(9.43)	
9-10	5 (5.32)	51.00(12.19)	
Special Courses			
Yes	18(14.63)	44.50(14.73)	*0.36
No	105(85.37)	42.05(11.00)	
Guidelines			
Yes	4(3.25)	47.25(11.30)	*0.46
No	119(96.75)	42.39(12.32)	

The mean of the student score (total score = 100 points) was 42.55 while SD was 12.27. The highest score observed in our study was 78 while the lowest score was 18 (Table 3).

**Table 3:** Mean, standard deviation, minimum, and maximum score for the total score and the four sections

Variable	Mean	Std Dev	Minimum	Maximum
Total Score (100 points)	42.55	12.27	18	78
Section 1. Blood Groups (5 points)	3.32	1.147	0	5
Section 2. Blood Products (30 points)	11.34	5.287	0	23
Section 3. Blood Transfusion (52 points)	21.49	7.617	4	47
Section 4. Infectious Diseases (13 points)	6.31	4.013	0	13

The dichotomous categorical variable "total score >50 or <50 " was related to the variables of Department/School (p = 0.00), year of study (p = 0.00), grade in hematology-immunology course (p = 0.00), and knowledge on transfusion issues (p = 0.02) (Table 4).

**Table 4:** Association of the dichotomous categorical variable (total scores >51 or <50) with independent variables p-value of χ2 test.

Variable	Percentage with Score > 50	p-value
Sex		
Male	32.70	0.11
Female	20.00	
Department/School		
Medicine	35.37	0.00
Biology	7.5	
Graduate of another School		
Yes	33.33	0.84
No	28.04	
Father's Education Level		
Senior High School	19.03	0.40
Technological Institute	33.33	
University Degree	29.03	
Mother's Education Level		
Senior High School	18.10	0.29
Technological Institute	29.17	
University Degree	32.08	
Father's Professional Status		
Employee	25.00	0.04
Civil Servant	15.91	
Self-employed	30.56	
Pensioner	52.94	
Unemployed	0.00	
Mother's Professional Status		
Employee	15.63	0.9
Civil Servant	25.53	
Self-employed	31.25	
Pensioner	60.00	
Unemployed	22.22	
Blood Donor		
Yes	24.44	0.76
No	26.92	
Knowledge on blood transfusion		
Poor	10.34	0.02
Little	25.42	
Good	41.94	
Very good	100.00	
Special Courses		
Yes	33.33	0.44
No	24.76	
Guidelines		
Yes	25.00	0.96
No	26.05	
Age		
<25 yo	27.43	0.91
>25 yo	25.00	
Grade in the Hematology course		
5-6,9	15.63	0.00
7-7,9	8.51	
8-8,9	60.00	
9-10	60.00	
Year of study		
3^rd^	5.88	0.00
4^th^	23.08	
5^th^	56.00	
6^th^	40.91	

From the results of the multiple regression model, the year of study, father's professional status, and grade in the hematology course remain statistically significant independent variables, while the department is no longer a statistically significant independent variable (Table 5).

**Table 5:** Results of multiple regression linear model with the total score

Variable	Coefficient b	95% Confidence Interval	p-value
(Constant)	55.25	(34.4, 76.1)	0,00
Department/School			
Medicine	-4.1	(-9.3, 1.0)	0.11
Biology	Ref	Ref	
Year of study			
3^rd^	8.3	(-13.9, -2.7)	0.00
4^th^	-6.1	(-12.6, 0.4)	0.07
5^th^	5.5	(-3.0, 14.0)	0.20
6^th^	Ref	Ref	
Father's Professional Status			
Employee	6.6	(-12.6, 25.8)	0.49
Civil Servant	6.8	(-11.6, 25.2)	0.46
Self-employed	5.9	(-12.5, 24.3)	0.53
Pensioner	21.5	(1.1, 41.9)	0.04
Unemployed	Ref	Ref	
Grade in the Hematology course			
5-6,9	-15.0	(-26.3, -3.8)	0.01
7-7,9	-17.1	(-27.7, -6.5)	0.00
8-8,9	0.0	(-12.0, 12.1)	1.00
9-10	Ref	Ref	

## Discussion

The results of the study showed a clear inefficiency in students' knowledge of hemotherapy issues. The largest gaps were observed in issues related to the production, maintenance and administration of blood products, transfusion, complications and their treatment, infectious disease transmission and their prevention. A similar study carried out in Iran by Aslani et al. (2013) to examine the knowledge of nurses on hemotherapy at the medical training centers at Shahrekord University, showed that 36.8% of the nurses achieved a score of 18.33/29, a score higher than the average. Regarding the knowledge of nurses about blood and its products, 21.4% of them had a good level of knowledge, 66.7% were rated on average, and 12% had poor knowledge. For the question "When would be the appropriate transfusion time after receiving the blood and its products from the blood bank", 81.2% of the students gave a wrong answer [19].

In the present study, in Section 3 (Transfusion), the mean was 21.49, the highest score 47/52, and the lowest score 4/52 (Table 2). For the question "What is the maximum time for the infusion of concentrated red blood cells", 14.53% answered correctly, while for the question "how long after their removal from the blood bank should platelets be transfused", 68, 91% gave the correct answer. The duration of transfusion of concentrated red cells must not exceed four hours, as the risk of developing Gram (-) bacteria increases, thus having a huge impact on the patient's health [20].

According to International data, the lack of knowledge about the maintenance of the blood and its products, which are due to be transfused from the blood bank and the duration of transfusion to the patient, can lead directly to a high risk of bacterial contamination (septicemia). It is the most common infectious complication of transfusions of hemotherapy as recorded by global organisms [20-21]. In Greece, 35 cases of serious bacteraemia after blood transfusion were recorded from 2001 to 2011 [22]. In our study, 64.23% responded correctly to the question "During the transfusion, the patient has a fever with or without shivering. What actions will you follow? " In a similar study carried out in the Democratic Republic of Congo by Kabinda et al. in 2014 (for a similar question), 90% were aware of the actions to be followed after such a reaction. In the same survey, only 3.1% responded correctly to questions related to accidental exposure of health professionals to blood and the knowledge of the existence of a protection system in hospital institutions [23-24].

In the study, in Section 4 (infectious diseases, total score=13 points), the mean was 6, 31, SD=4,013, lowest score= 0 and highest=13, as seen in Table 2. The WHO recommends mandatory control of all blood units for HIV ½, hepatitis B and C and syphilis, according to the requirements of the quality system [1]. During the period between 1997-2011, the transmission of HIV infection was recorded in two patients transfused with blood products of the same blood infected unit administered during the serological window period [22,25].

For the question "Have you read the blood transfusion guidelines", 3.25% answered yes, while 96.75% answered no. Newer technologies in education and learning, such as the combination of artificial intelligence, teaching models and learning models (cognition), can provide solutions to the problems of both the modern educational system of undergraduate and postgraduate studies and in health professionals' education through continuing education programs [26]. From the results, the knowledge about the treatment of hemotherapy provided to students of MD and DMB&G differ (44.63 vs. 38.25, respectively). It would be a good idea to have an identical training program for the students of all schools.

## Conclusions

The results of the study showed that the level of knowledge of the students of DM and DMB&G related to the transfusion of blood products and to transfusion-transmitted infectious diseases is insufficient. Findings may be due to the inadequate education of students by educational institutions. Clinical doctors require special knowledge and training on blood transfusion as a treatment, to be able to meet the demands of modern medicine. From the research data of the international bibliography, there is a clear need to disseminate sound scientific knowledge and integrated practice on issues related to hemotherapy. The correct and scientifically comprehensive knowledge of hemotherapy contributes to the safe transfusion of the patient and leads to the prevention of transmission of infectious diseases while reducing the likelihood of medical transfusion errors and the increase in morbidity and mortality. In addition to theoretical knowledge, practical lessons should be offered at blood donation centers. To acquire technical skills, clinical training should take place in real-life situations (low-risk cases and with the presence of a supervisor). In recent years, with the development of technology, Medical Education has introduced the use of simulation technologies borrowed from flight simulators in aviation. Transfusion medicine is proposed to be included in medical specialties, in the future, as a distinct specialty.
